# Supplementary figures and images for: Host specificity of gastrointestinal parasites in free-ranging sloths from Costa Rica
Source: PeerJ. 2025 May 8;13:e19408. doi: 10.7717/peerj.19408 (PMC12066103; doi:10.7717/peerj.19408)

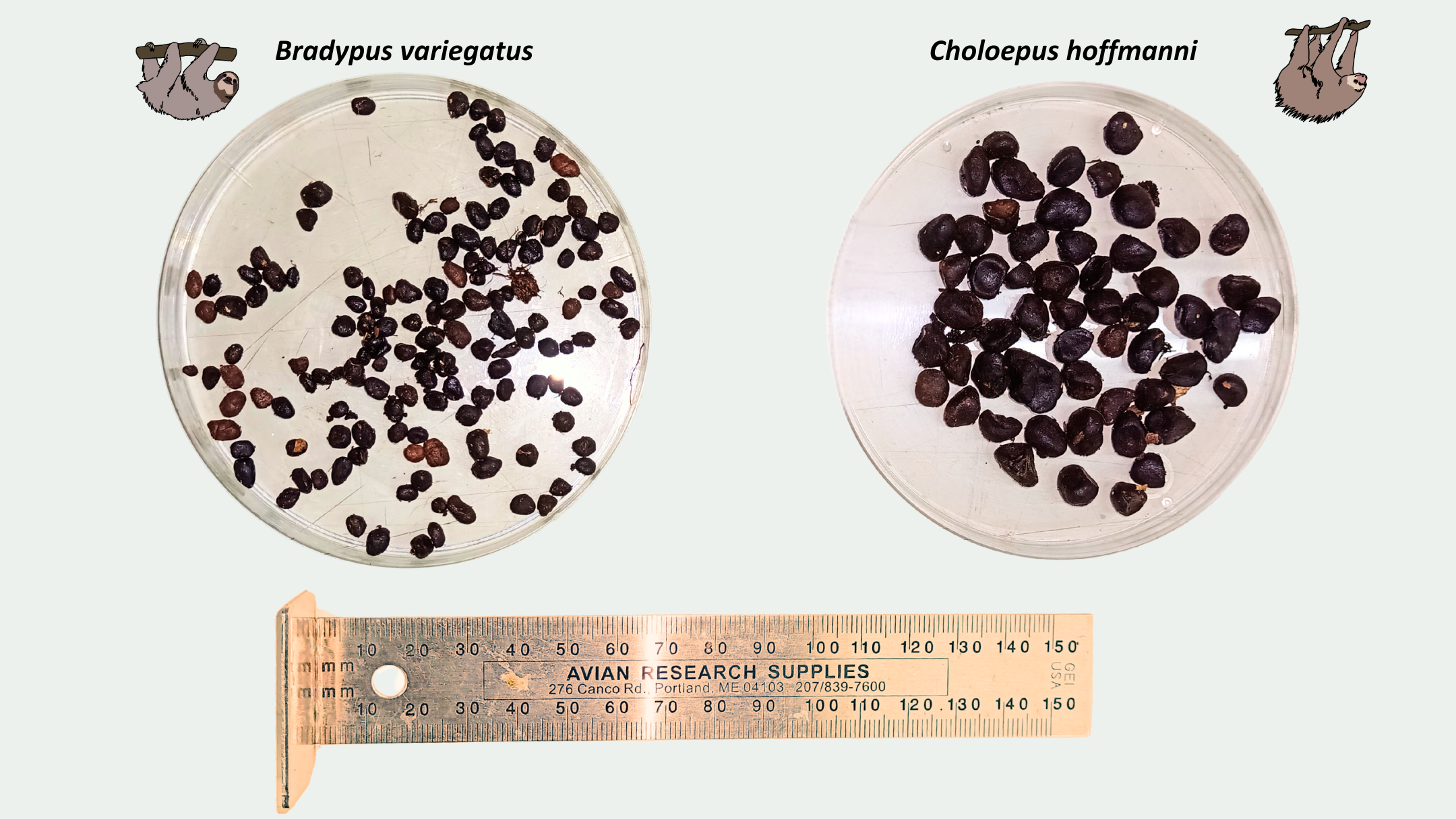

Supplement: Supplemental Information 1 — The scale is shown in millimeters. [file peerj-13-19408-s001.png]
