# Supplementary material for: Host specificity of gastrointestinal parasites in free-ranging sloths from Costa Rica
Source: PeerJ. 2025 May 8;13:e19408. doi: 10.7717/peerj.19408 (PMC12066103; doi:10.7717/peerj.19408)
Supplement: Supplemental Information 2 [file peerj-13-19408-s002.docx]

| **Host** | **Parasite species** | **Free or captive** | **Country** | **Citation** |
| --- | --- | --- | --- | --- |
| ***Bradypus tridactylus*** | *Leiuris leptocephalus* (Nematoda) | Free ranging | Brazil | Vaz & Pereira 1929 |
|  | *Leiuris pereirai* (Nematoda) | Free ranging | Brazil | Gomes & Vicente 1970 |
|  | *Leiuris vazipereirai* (Nematoda) | Free ranging | Brazil | Vaz & Pereira 1929 |
|  | *Paraleiuris locchii* (Nematoda) | Free ranging | Brazil | Vaz & Pereira 1929 |
|  | *Paraleiuris vazi* (Nematoda) | Free ranging | Brazil | Vicente & Gomes 1971 |
|  | *Giardia duodenalis* (Protozoa) | Captive | Brazil | Dos Reis et al., 2023 |
| ***Bradypus variegatus*** | *Leiuris leptocephalus* (Nematoda) | Free ranging | Costa Rica | Jiménez-Quirós & Brenes 1956 |
|  |  | Free ranging | Brazil | Werneck et al., 2008 |
|  | *Paraleiuris vazi* (Nematoda) | Captive | Brazil | Araujo et al., 2021 |
|  | *Paraleiuris locchii*  (Nematoda) | Captive | Brazil | Michel et al., 2017 |
|  | *Moniezia benedeni* (Cestoda) | Free ranging | Costa Rica | Flores Barroeta et al., 1958 |
|  | *Giardia duodenalis*  (Nematoda) | Captive | Panama | Hegner & Schumaker 1928 |
