# Supplementary material for: Host specificity of gastrointestinal parasites in free-ranging sloths from Costa Rica
Source: PeerJ. 2025 May 8;13:e19408. doi: 10.7717/peerj.19408 (PMC12066103; doi:10.7717/peerj.19408)
Supplement: Supplemental Information 4 [file peerj-13-19408-s004.docx]

**Appendix S1**

Studies of gastrointestinal parasites from sloths are limited, but nevertheless prior publications provide a basis for interpretation of our results. We reviewed all publications known to describe taxonomic identifications of gastrointestinal parasites from sloths, and found that reports were limited to only two host species of the genus *Bradypus*, specifically *B. variegatus* and *B. tridactylus*. There have been no prior descriptions of adult parasites from any of five the remaining sloth species (*Bradypus torquatus*, *B. crinitus*, *B. pygmaeus*, *Choloepus hoffmanni*, or *Choloepus didactylus*). In publications dating to 1928, a total of eight nematode species, one cestode, and one protozoan have been described in association with three-fingered sloths (Table S1). Coproparasitological studies are also limited, although two prior studies of sloths occurring in captivity and rural areas have reported observations of nematode, cestode, and protozoan eggs (Lainson & Shaw 1982; Sibaja-Morales et al., 2009). As with the current study, taxonomic identifications of potential parasite species based on observations of eggs can be challenging and thus the ability of these descriptions to support cross-study comparisons is limited.

Here, we provide a detailed review of taxonomic and morphological similarities between our results and the available literature. We err on the side of ensuring broadly accurate taxonomic descriptions, even in cases where taxonomic resolution is limited.

Five parasite morphotypes from *Bradypus variegatus* (Table 1; Figure 2).

1. Spirocercidae egg morphotype 1 (Figure 2a): This morphotype was found in one sample, and it resembles the Spirurida egg morphotype (Figure 2b), but its size is significantly smaller (Table 1). The family Spirocercidae has been reported in *Bradypus variegatus* and *B. tridactylus*, with *Leiurus* and *Paraleiurus* being the only described genera (Table S1). Egg sizes vary between the suite of host and Spirocercidae species found in sloths: *Paraleiurus vazi* (Vicente & Gomez, 1971) and *Leiurus pereirai* (Gomez & Vicente, 1970) exhibit similar measurements and larvated eggs.
2. Spirurida egg morphotype (Figure 2b): This morphotype was the most prevalent nematode egg in *B. variegatus* (Table 1). These eggs could potentially match prior descriptions of *Leiurus leptocephalus*, as adult worms from this species were reported in both Costa Rica and Brazil (Table S1; Jiménez-Quirós & Brenes, 1956; Werneck et al., 2005). Description of these eggs are based on gravid female worms included measurements of 56 by 22 μm (Vaz & Pereira, 1929), 33 by 13 μm (Jiménez-Quirós & Brenes, 1956), and an average of 44 by 24 μm (Werneck et al., 2005). This variability in egg measurements may be attributable to differences in female reproductive maturity, host species, and/or geographic variation.
3. Subuluridae egg morphotype (Figure 2c): This morphotype is being reported for the first time in *B. variegatus*. No prior publication of this family in association with sloths were identified in our review.
4. Ascaridida egg (Figure 2d): This morphotype is being reported for the first time in *B. variegatus*. No prior publication of this family in association with sloths were identified in our review.
5. Coccidia cyst morphotype (Figure 2e): Cysts of the protozoan Eimeridae (Apicomplexa) were reported in prior coprological surveys in sloth species (Lainson & Shaw 1982; Sibaja-Morales et al., 2009). It is not practical to provide a precise identification of protozoan species based on observations of cysts, further studies using specialized Protozoa techniques would be required to provide better taxonomic resolution.

Three parasite morphotypes from *Choloepus hoffmanni* (Table 1; Figure 3).

1. Anoplocephalidae morphotype (Figure 3a): This was the most prevalent helminth egg morphotype detected (Table 1). This family of Cestoda was reported in other coproparasitological studies in Costa Rica, though none reported egg sizes (Sibaja-Morales et al., 2009; Estrada-Rodríguez, 2007). The only Cestoda that has been described in sloths is *Moniezia benedini*, which was detected in *B. variegatus* (Flores-Barroeta et al., 1958).
2. Spirocercidae egg morphotype 2 (Figure 3b): This morphotype is being reported for the first time in *Choloepus* spp. No prior publication of this family in association with sloths were identified in our review.
3. Coccidia cyst morphotype 2 (Figure 3c): Cysts of the protozoan subfamily Eimeridae were reported in prior coprological surveys in sloths species (Lainson & Shaw 1982; Sibaja-Morales et al., 2009). It is not practical to provide a precise identification of protozoan species based on observations of cysts, further studies using specialized Protozoa techniques would be required to provide better taxonomic resolution.

Coproparasitological studies on free-ranging sloths that provide morphological characterizations of parasitic eggs and include images have served as key points of reference for our observations. In our review, we identified several inaccuracies in prior studies that are frequently cited in the relevant literature (Estrada, 2007; Sibaja-Morales et al., 2009). Estrada’s (2007) undergraduate thesis on sloths in cacao plantations and Sibaja-Morales’ (2009) peer-reviewed paper on captive sloths both report *Leiurus* and *Moniezia* specimens, but they lack illustrations, measurements, or voucher specimens for verification. The limited available data reflects the difficulty in assigning species to microscopic eggs and underscores the need for more robust methodologies that is not exclusive to sloths. Therefore, rather than assigning precise species or identifications that extend beyond the resolution of our data, we emphasize the need for further research using complementary techniques such as necropsies, systematic parasite collections, and molecular analyses to better identify wildlife parasites.

**References in the Supplementary Appendix**

Araujo, S. C. G. F., Branco, Ã., Lima, A. R. de, Pinheiro, R. H. da S., & Giese, E. G. (2021). Ocorrência de *Paraleiuris locchii* e *Paraleiuris vazi* em *Bradypus variegatus* no estado do Pará, Brasil. *Biota Amazônia (Biote Amazonie, Biota Amazonia, Amazonian Biota)*, *11*(1), Article 1. <https://doi.org/10.18561/2179-5746/biotaamazonia.v11n1p1-5>

Dos Reis, L. L., de Souza, L. S. S., Braga, F. C. D. O., Lima, D. C. D. S., Lima, N. A. D. S., Padinha, J. D. S., ... & Vicente, A. C. P. (2023). Zoonotic Giardia duodenalis assemblage A in northern sloth from Brazilian Amazon. Memórias do Instituto Oswaldo Cruz, 118, e230088. <https://doi.org/10.1590/0074-02760230088>

Estrada Rodríguez, A. C. (2007). Identificación de los parásitos de *Bradypus variegatus* y *Choloepus hoffmanni* (Xenarthra: Bradypodidae y Megalonychidae) en vida libre en Guácimo de Limón. Trabajo Final de Graduación. Universidad Nacional, Costa Rica.

Flores-Barroeta, L., Hidalgo-Escalante, E., & Brenes, R. R. (1958). Céstodos de Vertebrados VI. *Revista de Biología Tropical*, *6*(2), Article 2. <https://doi.org/10.15517/rev.biol.trop.1958.28965>

Gomes, D. C. & Vicente , J. J . , 1970 - Sôbre uma nova espécie do gênero *Leiuris* Leuckart, 1850 (Nematoda, Spiruroidea ). Atas Soe. Bio l. Rio de Janeiro , 14 ( 1/2): 11-12, 3 figs.

Hegner, R., & Schumaker, E. (1928). Some intestinal amoebae and flagellates from the chimpanzee, three-toed sloth, sheep and guinea-pig. The Journal of Parasitology, 15(1), 31-37.

Jiménez-Quirós, O., & Brenes, R. R. (1956). Presencia de *Leiurus leptocephalus* (Rudolphi, 1819) Leuckart, 1850 en *Bradypus griseus* (Gray, 1871) Allen, 1891. *Revista de Biología Tropical*, *4*(2), Article 2. <https://doi.org/10.15517/rbt.v4i2.28896>

Lainson, R., & Shaw, J. J. (1982). Coccidia of Brazilian edentates: *Eimeria cyclopei* n. sp. from the silky anteater, *Cyclopes didactylus* (Linn.) and *Eimeria choloepi* n. sp. from the two-toed sloth, *Choloepus didactylus* (Linn.). Systematic Parasitology, 4(3), 269-278.

Michel, A. F. R. M., Silva, F. L., Barbosa, F. S., Carvalho, T. F. de, Pinto, J. M. S., & Santos, R. L. (2017). Ulcerative and necrotizing gastritis in a captive sloth (*Bradypus variegatus* , Xenarthra, Bradypodidae) due to severe parasitism with *Paraleiuris locchii* (Nematoda, Spirocercidae). Journal of Zoo and Wildlife Medicine, 48(1), 255-259. <https://doi.org/10.1638/2016-0135.1>

Vaz, Z. & Pereira, C. Arduenninae de *Bradypus tridactylus* L. Bol. Biol., v.15-16, p.1-15, 1929.

Sibaja-Morales, K. D., Oliveira, J. B. de, Rocha, A. E. J., Gamboa, J. H., Gamboa, J. P., Murillo, F. A., Sandí, J., Nuñez, Y., & Baldi, M. (2009). Gastrointestinal Parasites and Ectoparasites of *Bradypus variegatus* and *Choloepus hoffmanni* sloths in Captivity from Costa Rica. Journal of Zoo and Wildlife Medicine, 40(1), 86-90. <https://doi.org/10.1638/2008-0036.1>

Vicente, J. J., & Gomes, D. C. (1971). Sôbre um Nematódeo spirurídeo parasito de *Bradypus Tridactylus* L. Memórias do Instituto Oswaldo Cruz, 69, 71-73.
